# Effects of Message Framing on Cancer Prevention and Detection Behaviors, Intentions, and Attitudes: Systematic Review and Meta-analysis

**DOI:** 10.2196/27634

**Published:** 2021-09-16

**Authors:** Abidan Ainiwaer, Shuai Zhang, Xiayiabasi Ainiwaer, Feicheng Ma

**Affiliations:** 1 School of Information Management, Wuhan University Wuhan China; 2 School of Public Health, Fudan University Shanghai China

**Keywords:** gain framing, loss framing, attitude, intention, behaviors, cancer prevention, cancer detection

## Abstract

**Background:**

With the increasing health care burden of cancer, public health organizations are increasingly emphasizing the importance of calling people to engage in long-term prevention and periodical detection. How to best deliver behavioral recommendations and health outcomes in messaging is an important issue.

**Objective:**

This study aims to disaggregate the effects of gain-framed and loss-framed messages on cancer prevention and detection behaviors and intentions and attitudes, which has the potential to inform cancer control programs.

**Methods:**

A search of three electronic databases (Web of Science, Scopus, and PubMed) was conducted for studies published between January 2000 and December 2020. After a good agreement achieved on a sample by two authors, the article selection (κ=0.8356), quality assessment (κ=0.8137), and data extraction (κ=0.9804) were mainly performed by one author. The standardized mean difference (attitude and intention) and the odds ratio (behaviors) were calculated to evaluate the effectiveness of message framing (gain-framed message and loss-framed message). Calculations were conducted, and figures were produced by Review Manager 5.3.

**Results:**

The title and abstract of 168 unique citations were scanned, of which 53 were included for a full-text review. A total of 24 randomized controlled trials were included, predominantly examining message framing on cancer prevention and detection behavior change interventions. There were 9 studies that used attitude to predict message framing effect and 16 studies that used intention, whereas 6 studies used behavior to examine the message framing effect directly. The use of loss-framed messages improved cancer detection behavior (OR 0.76, 95% CI 0.64-0.90; *P*=.001), and the results from subgroup analysis indicated that the effect would be weak with time. No effect of framing was found when effectiveness was assessed by attitudes (prevention: SMD=0.02, 95% CI –0.13 to 0.17; *P*=.79; detection: SMD=–0.05, 95% CI –0.15 to 0.05; *P*=.32) or intentions (prevention: SMD=–0.05, 95% CI –0.19 to 0.09; *P*=.48; detection: SMD=0.02, 95% CI –0.26 to 0.29; *P*=.92) among studies encouraging cancer prevention and cancer detection.

**Conclusions:**

Research has shown that it is almost impossible to change people's attitudes or intentions about cancer prevention and detection with a gain-framed or loss-framed message. However, loss-framed messages have achieved preliminary success in persuading people to adopt cancer detection behaviors. Future studies could improve the intervention design to achieve better intervention effectiveness.

## Introduction

### Background

Cancer accounts for 1 in 6 deaths globally. The number of cancer cases worldwide may increase by 60%, especially in low-income and middle-income countries where the increase may be as high as 81%, as estimated by the latest World Cancer Report [1]. This trend will undoubtedly lead to socioeconomic pressure and a shortage of medical resources. The current clinical research on cancer medicine mainly focuses on finding the cause of cancer and preventing the spread of cancer cells in the early stage [2,3]. However, these findings can be helpful only if people are sufficiently aware of adopting healthy behavior (ie, prevention and detection). This is a unique form of a health crisis that requires intensive communicative efforts. Therefore, messages need to be carefully designed in cancer communication to achieve positive health outcomes.

Message framing is an effective technique to change health behavior [4-10]. Health messaging attempts to change people's attitudes, intentions, or behaviors toward a specific health topic by emphasizing the expected benefits of undergoing specific health behaviors (ie, gain-framed messaging) or the possible loss if specific health behaviors are not done (ie, loss-framed messaging), to persuade people to follow healthy guidelines. According to the theory of reasoned action, attitude (or intention) is an essential direct predictor of behavior [11-13]. Moreover, attitudes, intentions, and behaviors are the standard measures of the effectiveness of health messages [4,14].

Health behaviors include disease prevention and detection behavior [4,6]. The former aims to avoid illness or deterioration, while the latter aims to reflect the presence or absence of risk. Gain-framed messaging fulfills the promise of a safer and more certain disease prevention measure in terms of disease prevention, so it is more effective than loss-framed messaging [4,15]. O’Keefe and Jensen [16] also showed the advantage of the gain-framed messaging in disease prevention, but they indicate that it does not apply to skin cancer [17,18]. On the other hand, in terms of disease detection, it was thought that the loss-framed messaging showed a higher persuasive effect [4,15]. However, the meta-analysis results show no significant difference between gain and loss framing [6,16]. Thus, apart from demonstrating the contribution of loss-framed messaging to breast cancer detection [17], there has been no definitive conclusion regarding cancer and message framing.

### Objectives

The concept of message framing is an essential strategy in health promotion, but research on how to optimally frame cancer prevention and detection messaging is scarce. Furthermore, while the effectiveness of message framing in cancer prevention and detection remains unclear, there is still an increasing number of related studies that combine message framing with other variables (ie, color) to verify the strengthening or weakening of the framing effect. Therefore, a thorough examination of the effectiveness of the message framing in the context of cancer is needed.

This review mainly aims to systematically summarize the characteristics of the relevant intervention studies and then pool the effect sizes from the relevant studies to quantify the effects of those interventions on attitude, intention, and behavior change. The findings of this review can provide recommendations for researchers and clinicians to design effective messages in cancer communication.

## Methods

### Search Strategies

**Figure 1:**
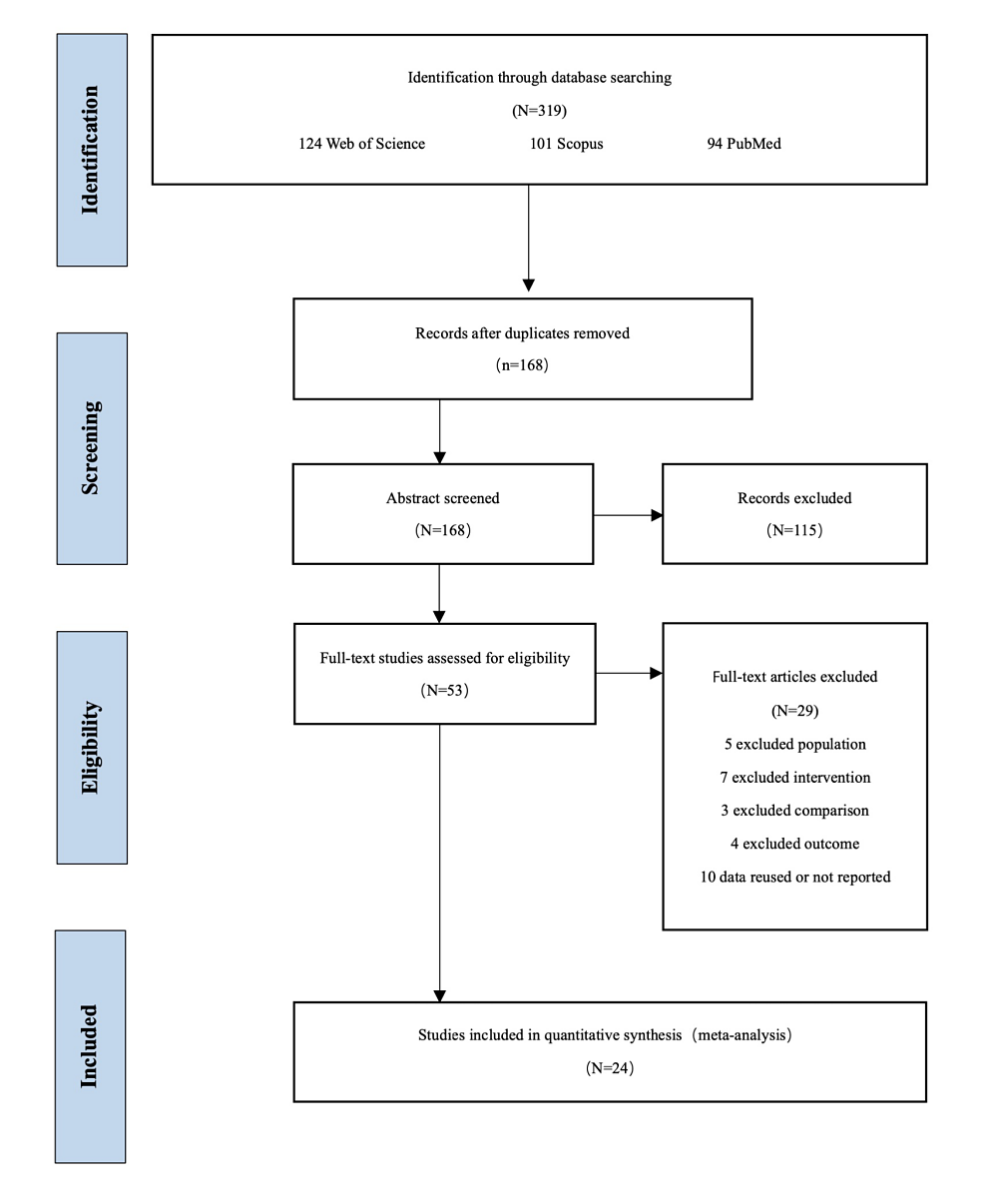
Preferred reporting items for systematic reviews and meta-analysis flowchart.

This review was conducted and is reported according to the PRISMA (preferred reporting items for systematic reviews and meta-analyses) guidelines. As mentioned in the initial registration (registration ID CRD42021252658), a series of structured electronic searches were performed in three English databases, including Web of Science, Scopus, and PubMed, focusing on the effectiveness of message framing related to a cancer topic and dated from January 1, 2000, to December 31, 2020. The procedures guiding article inclusion are presented in the flow chart in Figure 1. Example search terms are as follows: goal fram*, loss fram*, message fram*, goal fram*, health message, health infor*AND attitude, intention, behavio*, behavio*intention, behavio* chang* AND cancer, screen*, prevention, detection, AND specific validated database filters for randomized controlled trials

All articles identified in the search strategy were exported into reference management software (version 2.48.0; Mendeley) for duplicate checking and further screening. The reference lists of eligible articles were further reviewed to identify other relevant studies. Relevant reviews that emerged from the search strategy were checked for any additional studies. Grey literature (ie, working papers, unpublished studies, conference proceedings or abstracts, and dissertations) was not considered eligible. The preliminary retrieval process was taken as the total literature volume combining the reviews independently completed by two authors. The authors selected a sample of eligible studies after achieving good agreement (κ=0.8356), with the remainder being selected by one author.

### Study Inclusion and Exclusion Criteria

Studies were included if they met the following criteria: (1) participants (not cancer patient or survivors) of both genders and any age range exposed to either the loss-framed or the gain-framed cancer prevention or detection (not treatment) message in experimental research; (2) interventions that have been delivered via either emphasizing the expected benefits of taking specific health behaviors (gain-framed message) or the possible loss of not taking specific health behaviors (loss-framed message); (3) the gain-framed and loss-framed message must come in pairs as a comparison and related to a specific cancer topic, not cancer in general; (4) the effectiveness of cancer-related messages was measured in terms of change in attitude (by scale), intention (by scale), and behavior (by yes or no), and the analysis of primary data was reported, and sufficient quantitative data was provided for estimating the total effect size. In addition, only randomized controlled trials (RCTs) were included. Finally, published studies that repeatedly used the same data were excluded, leaving only one study that reported the most complete results.

### Data Extraction

Data from a sample of eligible studies was extracted by two authors, and after they achieved good agreement (κ=0.9804), the remainder was extracted by one author. The main framework of the extraction criteria was drawn by the authors altogether. The following information was extracted: (1) basic study characteristics including the first author, publication date, country, and funding of the study; (2) participant characteristics including sample size, age, ethnic, and gender ratio (female); (3) intervention characteristics including cancer type, research setting (lab or not), message contents, message resources, message delivery channel, intervention duration, and underpinning theories; (4) outcome measures including measurement of the outcomes; and (5) main results including intervention completion ratio and converted effect size (standardized mean difference [SMD] and odds ratio [OR]).

### Bias Assessments

The risk of bias for RCTs was independently assessed by two authors (κ=0.8137) using the Cochrane Collaboration tool for assessing bias [19]. Differences of opinion were discussed and agreed upon by the two authors.

### Strategy for Data Synthesis and Meta-Analysis

All meta-analyses were performed with Review Manager 5.3 (version 5.3; The Cochrane Collaboration) [20]. This study performed meta-analyses on the three message effectiveness indicators (attitude, intention, and behavior). Among them, for the two continuous variables (attitude and intention), the SMD of the gain-frame (as the intervention group) and the loss-frame (as the control group) are calculated to represent an effect size. Moreover, two subgroups of cancer prevention and cancer detection were designed separately and analyzed under each indicator. For the two-category variable (behavior), the OR of the gain-frame (as the intervention group) and the loss-frame (as the control group) are calculated to represent an effect size. In addition, two subgroups of cancer detection within 6 months (<6 months) or more than 6 months (>6 months) after the experiment were designed for comparison and analysis of the impact of the timing of the framing effect on cancer detection behavior. All meta-analyses used random-effects models. I^2^ statistics are used to determine heterogeneity and measure the degree of inconsistency. I^2^ values are 25%, 50%, and 75%, corresponding to low, medium, and high levels of heterogeneity, respectively. For high heterogeneity, the source of heterogeneity was investigated by conducting a subgroup analysis to explore potential moderators and demonstrate why heterogeneity existed. We used the funnel plot to observe whether there is publication bias and used the Egger regression line to confirm possible publication bias further.

## Results

### Study Characteristics

The literature search identified a total of 319 studies extracted by two authors. After deduplication, a total of 168 studies’ titles and abstracts were screened, with 53 remaining for full-text screening. Among those, 24 articles met the inclusion criteria, and 4 studies met the inclusion criteria but did not meet the exclusion criteria. The authors attempted to obtain the research data of 8 studies that met the first two inclusion criteria but failed to provide usable data by contacting their corresponding authors via email and received 2 replies [21,22]. Due to the oversize issue, the main content of each study is summarized in Multimedia Appendix 1. Of the 24 studies used in the final analysis, 13 were conducted by the United States [21,23-34], 2 by the United Kingdom [35,36], South Korea [22,37], and China [38,39], and 1 by Australia [40], Ireland [41], Italy [42], Singapore [43], and South Africa [44]. Among the studies used, 5 were funded by the government or institute grant [22,23,25,26,38], 2 by the institute and the center [24,29], 2 by the institute and universities [30,34], 2 by universities [28,37], and 1 by the health charity and behavioral insights project [35]. The remaining 12 studies did not report receiving any funding [21,27,31-33,36,39-44].

The total sample size was 11,637, ranging from n=85 (intervention group) to n=752 (control group). The participants’ average age ranged from 12 to 91 years. Because some cancers are associated with a particular sex, 2 studies notably recruited all-male participants [32,42], while another 10 studies recruited all-female participants [21,23,24,26,30,34,35,37,39,44]. Also, there was a range of different cancer topics targeted during the study interventions, including 9 studies for skin cancer [22,25,27,31,33,36,40,41,45], 2 for colorectal cancer [28,38], and 1 for lung cancer [29].

Most of the experiments were conducted online, through email [27-29,40,43,44], a phone call [23] or text message [35], and other methods like webpage URLs [31,37]. There are also a small number of experiments that were done in a laboratory setting using traditional printed materials [21,22,25,32,33,41,42,45]. Messages were delivered in a variety of ways, including text only [27,28,36,44], text plus images or graphics [26,29,38,40], and video [24,30,34,39,43]. The majority of studies were based on prospect theory [21,22,24,27,29,32,34,37,41,43-45], and some of them used theory of planned (or reasoned) behavior [28,31,38,39], self-affirmation theory [36], and the health belief model [30,42]. There are also some studies that did not use theories or models [23,25,35]. The majority of studies focused on attitude [28,31,32,37-40,42,43] and intention [21,22,25-29,33,36,37,39-43,45], whereas 6 studies used behavior to examine the message framing effect directly [23,24,30,34,35,44].

### Bias and Heterogeneity Assessments

**Figure 2:**
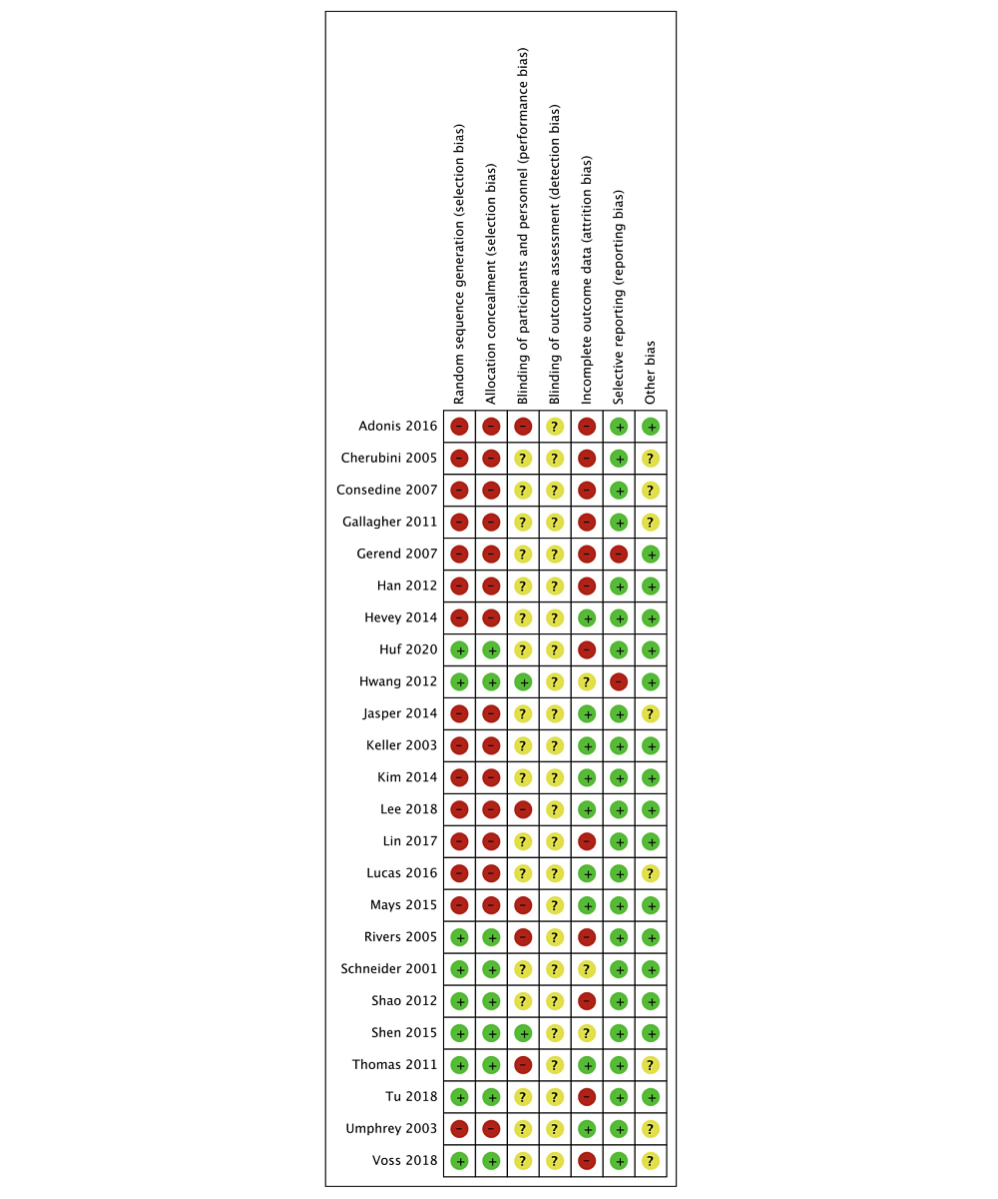
Risk of bias summary of the individual studies (k=24).

**Figure 3:**
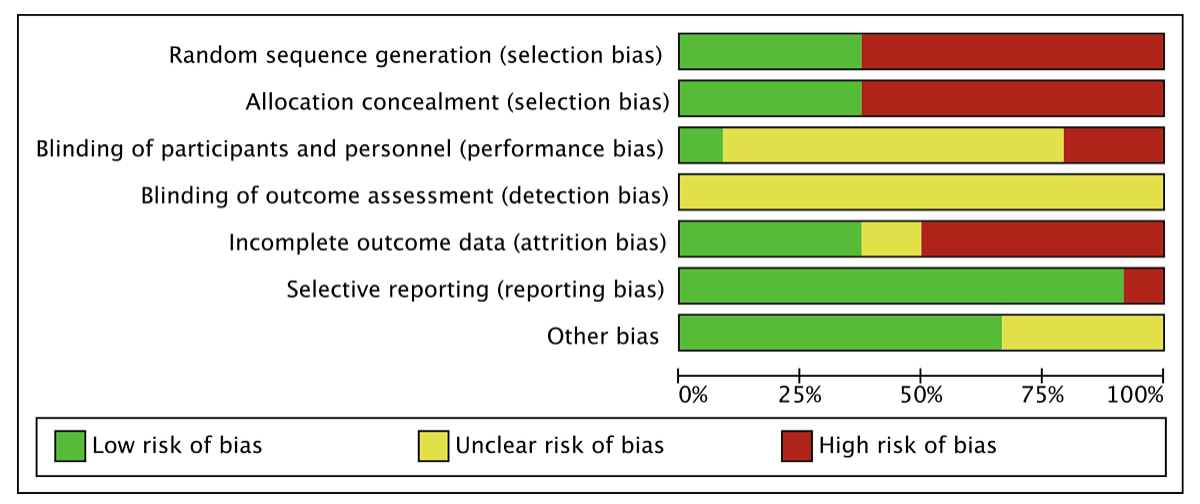
Risk of bias graph.

As shown in Figures 2 and 3, the risk of bias assessment indicated that the included articles were relatively high in quality. For the publication bias examination, the funnel plot was used for preliminary identification and the Egger regression line to confirm the possible publication bias further.

### Intervention Effectiveness

In the following sections, the synthesized results regarding attitude-related, intention-related, and behavior-related outcomes are introduced individually. The main results are visualized in the forest plot.

#### Attitude-Related Outcomes

Attitudes are commonly defined as “a psychological tendency that is expressed by evaluating a particular entity with some degree of favor or disfavor.” In this study, an attitude refers to the tendency towards engaging in a particular behavior or object targeted by a study's intervention.

**Figure 4:**
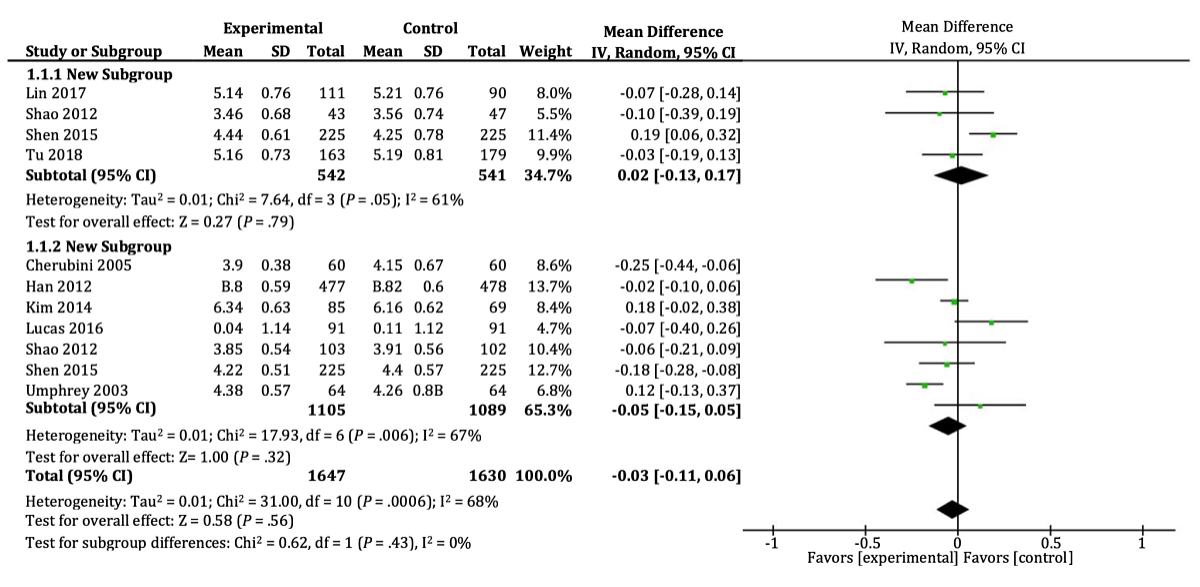
Forest plots of cancer detection attitude and cancer prevention attitude.

The attitude was used to indicate the persuasiveness of cancer-related messages in 9 studies. The relevant data involved a total of 3277 participants, including 1647 in the gain group and 1630 in the loss group. As seen in Figure 4, no significant difference was found between the two groups in either cancer prevention attitude (SMD=0.02, 95% CI –0.13 to 0.17; *P*=.79) or cancer detection attitude (SMD=–0.05, 95% CI –0.15 to 0.05; *P*=.32). There was medium heterogeneity (I^2^=68%; *P*<.001) across the trials.

The medium heterogeneity can be attributed to the study by Kim [43] and Shen [31]. Participants of both genders were involved in Kim [43], which focused on breast cancer, while the other studies all invited gender-specific participants who were more likely to be affected by the cancer of interest. In addition, Shen [31] invited students to participate in the experiment, and women accounted for 69% of participants. As undergraduates may be more sensitive to information about skin cancer because they are more focused on appearance rather than because they perceive health risks, excluding these 2 studies did decrease the heterogeneity (I^2^=0%, *P*=.11), but no significant difference still existed between the gain and loss groups in either cancer prevention intention (SMD=–0.05, 95% CI –0.17 to 0.06; *P*=.37) or cancer detection intention (SMD=–0.05, 95% CI –0.15 to 0.04; *P*=.26).

The funnel plot (Multimedia Appendix 2) demonstrates relative symmetry for the studies of cancer detection or prevention attitude, which suggests no publication bias existed and the above analysis was reliable.

#### Intention-Related Outcomes

Intention refers to a state of wanting, planning, or expecting to act in a given way. It can be general (ie, intending to mammogram) or specific (i.e., intending to mammogram in 3 months). In this study, the difference between intentions and other related cognitions such as expectations or willingness was not distinguished.

**Figure 5:**
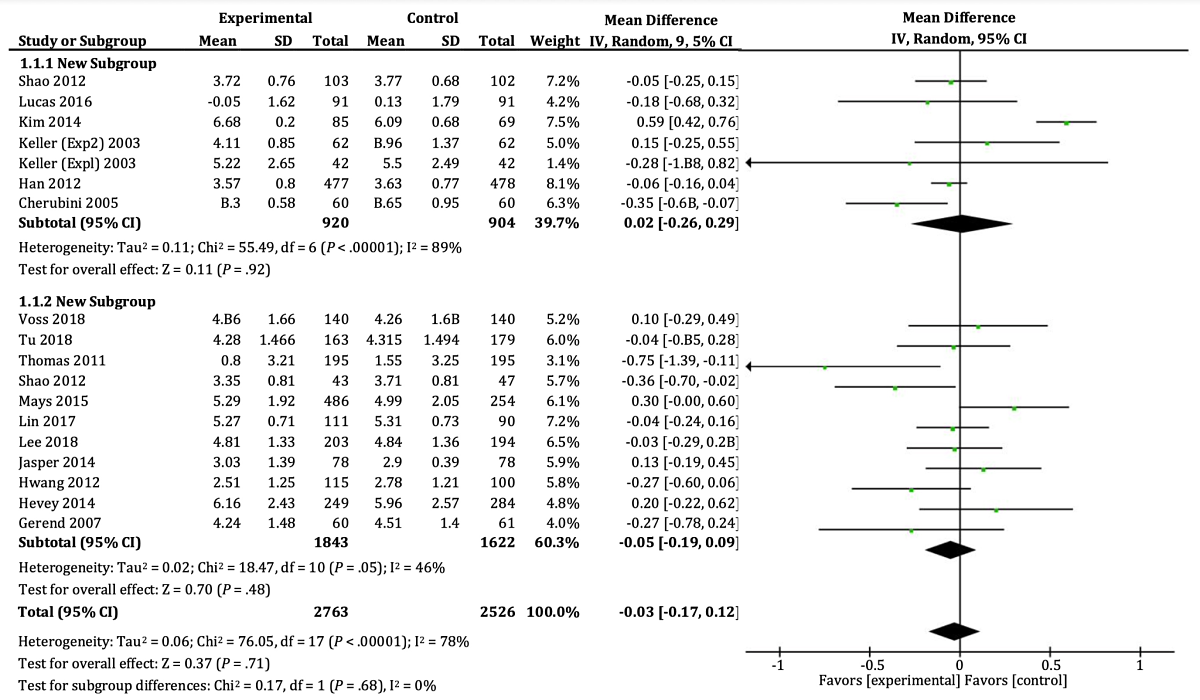
Forest plots of cancer detection intention and cancer prevention intention.

The intention was used to indicate the persuasiveness of cancer-related messages in 16 studies. The relevant data involved a total of 5289 participants, including 2763 in the gain group and 2526 in the loss group. As shown in Figure 5, no significant difference was found between the 2 groups in either cancer prevention intention (SMD=–0.05, 95% CI –0.19 to 0.09; *P*=.48) or cancer detection intention (SMD=0.02, 95% CI –0.26 to 0.29; *P*=.92). There was large heterogeneity (I^2^=78%; *P*<.001) across the trials.

Because the large heterogeneity can also be attributed to the study by Kim [43], excluding this study did decrease the heterogeneity (I^2^=34%, *P*=.09). In addition, the heterogeneity is due to the gender characteristics of the participants, as mentioned above. However, there is still no significant difference between the gain and loss groups in either cancer prevention intention (SMD=–0.05, 95% CI –0.19 to 0.09; *P*=.48) or cancer detection intention (SMD=–0.08, 95% CI –0.17 to 0.01; *P*=.08).

The funnel plot (Multimedia Appendix 3) demonstrates relative symmetry for the studies of cancer detection or prevention intention except for Kim [43], which suggests that there existed no publication bias after excluding this study.

#### Behavior-Related Outcomes

In most cases, behavioral measures are obtained by researchers using self-reported data from participants through follow-up surveys. However, it does not rule out the existence of special access to behavioral data (eg, a health insurance database record).

**Figure 6:**
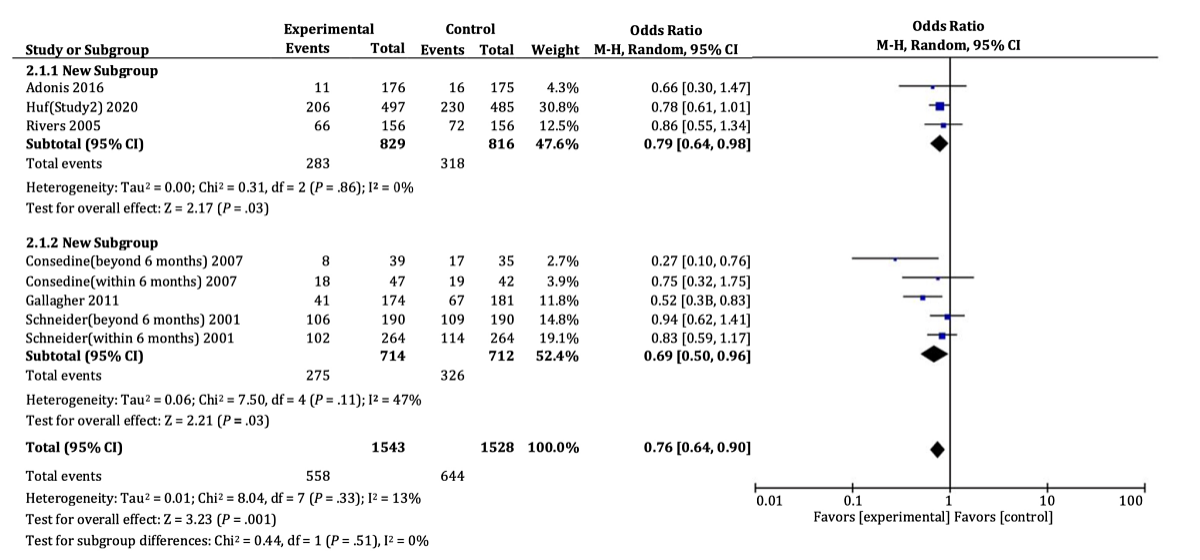
Forest plots of cervical cancer detection behavior and breast cancer detection behavior.

Behavior was used to indicate the persuasiveness of messages about cancer detection in 6 studies. The relevant data involved a total of 3071 participants, including 1543 in the gain group and 1528 in the loss group. It should be mentioned that cancer detection behavior was measured twice in Schneider et al [30] and Consedine et al [23]. According to Figure 6, loss-framed messages were significantly more likely than gain-framed ones to persuade people into engaging in both cervical cancer detection behavior (OR 0.79, 95% CI 0.64-0.98; *P*=.03) and breast cancer detection behavior (OR 0.69, 95% CI 0.50-0.96; *P*=.03), with no heterogeneity (I^2^=13%; *P*=.33).

There was often a time interval between cancer detection behavior and the experiment in which the participants read the framed messages. For the five measurements of cancer detection behavior that were performed within 6 months after the experiments, loss-framed messages were still significantly more persuasive than gain-framed ones (OR 0.76, 95% CI 0.61-0.95; *P*=.01), with no heterogeneity (I^2^=0%; *P*=.82). However, the intervention of loss framing became ineffective beyond 6 months (OR 0.73, 95% CI 0.49-1.11; *P*=.14), with medium heterogeneity (I^2^=59%; *P*=.09).

The funnel plot (Multimedia Appendix 4) demonstrates asymmetry for the studies of cancer detection behavior, from which one cannot tell whether publication bias existed. However, the Egger linear regression analysis suggests there was no publication bias (*P*=.09).

#### Relationships Between Behavior and Attitude or Intention

Given that significant results were obtained for cervical or breast cancer detection behavior, 4 studies of detection attitude or intention focusing on either of these two cancers were extracted to examine the relationships between behavior and attitude or intention. Excluding Kim [43] as a source of heterogeneity, the relevant data involved a total of 1284 participants, including 641 in the gain group and 643 in the loss group. No significant difference was found between the two groups in cancer detection attitude or intention (SMD=–0.05, 95% CI –0.14 to 0.03; *P*=.20), with no heterogeneity (I^2^=0.0%; *P*=.76). That is, as far as cervical and breast cancers are concerned, cancer detection behavior was not necessarily preceded by cancer detection attitude or intention. The funnel plot demonstrates relative symmetry for the studies of cancer detection attitude or intention, which suggests no publication bias existed, and the above analysis was reliable.

## Discussion

### Principal Findings

This study meta-analyzed 24 empirical studies on the persuasive effect of message framing in cancer prevention and detection. By subdividing the convincing effect into three dimensions, attitude, intention, and behavior, the results show that gain-framed and loss-framed messages have no significant differences in people's attitudes and intentions to change their cancer prevention or detection behavior. However, for cancer detection behavior, especially breast and cervical cancer detection, loss-framed messaging is more persuasive than gain-framed messaging, and this effect gradually weakens over time.

This meta-analysis did not identify any advantages of gain-framed messaging. Previous studies have proposed that gain-framed messaging is more persuasive than loss-framed in disease prevention, especially preventative behavior [6,16]. Since no relevant data on prevention behavior in the cancer-related literature are included in this meta-analysis, it cannot further verify whether the predecessors' benefits in disease prevention behavior can be equally applicable to the cancer topic. The results also negated the comparative advantage of loss framing in disease detection. There are several possible reasons for this contradictory conclusion. First, the previous meta-analysis did not distinguish between the three measurement indicators, so the results were affected by a large number of measurement indicators [16]. Second, the amount of cancer-related literature included in the previous meta-analysis is small, so the conclusions drawn do not apply to all cancer topics [6]. Therefore, it is necessary to verify whether these existing conclusions can be applied to all health topics based on previous studies. Moreover, because of different health topics, the focus also varies. Some emphasize daily prevention (such as using sunscreen), while some require regular detection (such as a colonoscopy).

This meta-analysis also supports some of the results of previous studies. Neither gain-framed nor loss-framed messaging can exert the framing effect of changing people's attitudes or intentions [6]. Our meta-analysis includes multiple cancer themes, and it also expands the positive impact of the loss framing found in breast cancer detection [46].

One interesting finding of this meta-analysis is that the message framing effect is time-limited. Others have questioned the duration of the framing effect in explaining the declining return rate [34]. On the other hand, this meta-analysis evaluates a larger scale of data and finds that the framing effect diminishes over time. Therefore, future research could further investigate how often health information stimulation can exert the framing effect and determine the effect of repeating the same framing.

Another notable result of this study is the disconnect between people's attitudes, intentions, and behavior. When reorganizing data related to attitudes and intention for breast and cervical cancer prevention and detection, these results also show that attitudes and intentions cannot effectively predict behavior, indicating that there may indeed be a disconnect between people's attitudes, intentions, and behavior. This conclusion has also been confirmed by previous studies [6,47]. The ultimate purpose of any framed message, but especially a health message, is to promote a specific behavior. In the research process, paying too much attention to the influence of other variables besides behavior may outweigh the gains. Further verification is worthwhile to measure whether subjective attitudes and intentions to target behaviors can replace or predict the subsequent behavior after reading the framed message. The exploration in this area cannot stop at testing latent variables such as attitude and intention, and it should return its focus to “behavior” itself [48].

### Limitations

As with all meta-analyses, this study was limited by the included literature measurement indicators and provided data. For example, the measurement data of attitude and intention included in the literature far exceed behavior-related data. Cancer detection behavior data were obtained only due to the lack of actual behavioral data related to cancer prevention. Also, the data are limited to two types of cancer (breast cancer and cervical cancer). Its generalizability also needs to be further verified based on more relevant research. Due to the low email response rate (2 out of 8), this meta-analysis excluded some studies meeting the inclusion criteria.

### Conclusions

This meta-analysis shows that gain framing and loss framing have no significant difference in attitudes and intentions about cancer prevention and detection. On the other hand, loss framing can promote breast cancer and cervical cancer detection better than gain framing. However, the effect of the loss framing gradually weakens over time. Therefore, when constructing a message to promote cancer detection, more considerations can be given to loss framing’s promised short-term effects. People can be screened for related cancers quickly by emphasizing the possible risk of not performing cancer detection.

## Appendix

Multimedia Appendix 1 Detailed records of eligible studies.

Multimedia Appendix 2 Funnel plot depicting the publication bias of attitude-related studies.

Multimedia Appendix 3 Funnel plot depicting the publication bias of intention-related studies.

Multimedia Appendix 4 Funnel plot depicting the publication bias of behavior-related studies.

## Abbreviations

OR: odds ratio
PRISMA: preferred reporting items for systematic reviews and meta-analyses
RCT: randomized controlled trial
SMD: standardized mean difference

## References

[1] Wild C, Weiderpass E, Bernard WS World Cancer Report: Cancer research for Cancer Prevention, 2020. International Agency of Research on Cancer, World Health Organization.

[2] Anderson RL, Balasas T, Callaghan J, Coombes RC, Evans J, Hall JA, Kinrade S, Jones D, Jones PS, Jones R, Marshall JF, Panico MB, Shaw JA, Steeg PS, Sullivan M, Tong W, Westwell AD, Ritchie JWA, Cancer Research UKCancer Therapeutics CRC Australia Metastasis Working Group (2019). A framework for the development of effective anti-metastatic agents. Nat Rev Clin Oncol.

[3] Piberger AL, Bowry A, Kelly RDW, Walker AK, González-Acosta Daniel, Bailey LJ, Doherty AJ, Méndez Juan, Morris JR, Bryant HE, Petermann E (2020). PrimPol-dependent single-stranded gap formation mediates homologous recombination at bulky DNA adducts. Nat Commun.

[4] Rothman AJ, Salovey P (1997). Shaping perceptions to motivate healthy behavior: the role of message framing. Psychol Bull.

[5] Akl EA, Oxman AD, Herrin J, Vist GE, Terrenato I, Sperati F, Costiniuk C, Blank D, Schünemann Holger (2011). Framing of health information messages. Cochrane Database Syst Rev.

[6] Gallagher KM, Updegraff JA (2012). Health message framing effects on attitudes, intentions, and behavior: a meta-analytic review. Ann Behav Med.

[7] Graham Amanda L, Fang Ye, Moreno Jose L, Streiff Shawn L, Villegas Jorge, Muñoz Ricardo F, Tercyak Kenneth P, Mandelblatt Jeanne S, Vallone Donna M (2012). Online advertising to reach and recruit Latino smokers to an internet cessation program: impact and costs. J Med Internet Res.

[8] Jiang T, Wu X, Chen Y, Wang Y (2021). The effects of message framing on online health headline selection of female users: A moderation of approach/avoidance motivation. Int J Med Inform.

[9] Niemiec R, Jones MS, Mertens A, Dillard C (2021). The effectiveness of COVID-related message framing on public beliefs and behaviors related to plant-based diets. Appetite.

[10] Xu X, Yang M, Zhao YC, Zhu Q (2020). Effects of message framing and evidence type on health information behavior: the case of promoting HPV vaccination. AJIM.

[11] Triandis HC (1980). Values, attitudes, and interpersonal behavior. Nebr Symp Motiv.

[12] Vezich I, Katzman P, Ames D, Falk E, Lieberman M (2017). Modulating the neural bases of persuasion: why/how, gain/loss, and users/non-users. Soc Cogn Affect Neurosci.

[13] Matkovic J, Clemens KS, Faasse K, Geers AL (2021). Handwashing Message Type Predicts Behavioral Intentions in the United States at the Beginning of the Global COVID-19 Pandemic. Front Public Health.

[14] Ballard AM, Davis A, Hoffner CA (2021). The Impact of Health Narratives on Persuasion in African American Women: A Systematic Review and Meta-Analysis. Health Commun.

[15] Rothman A, Bartels R, Wlaschin J, Salovey P (2006). The strategic use of gain- and loss-framed messages to promote healthy behavior: How theory can inform practice. Journal of Communication.

[16] O'keefe DJ, Jensen JD (2006). Chapter 1: The Advantages of Compliance or the Disadvantages of Noncompliance? A Meta-Analytic Review of the Relative Persuasive Effectiveness of Gain-Framed and Loss-Framed Messages. Communication Yearbook.

[17] O'Keefe DJ, Jensen JD (2007). The relative persuasiveness of gain-framed and loss-framed messages for encouraging disease prevention behaviors: a meta-analytic review. J Health Commun.

[18] O'Keefe DJ, Wu D (2012). Gain-framed messages do not motivate sun protection: a meta-analytic review of randomized trials comparing gain-framed and loss-framed appeals for promoting skin cancer prevention. Int J Environ Res Public Health.

[19] Higgins JPT, Altman DG, Gøtzsche PC, Jüni P, Moher D, Oxman AD, Savovic J, Schulz KF, Weeks L, Sterne JAC, Cochrane BMG, Cochrane SMG (2011). The Cochrane Collaboration's tool for assessing risk of bias in randomised trials. BMJ.

[20] Sterne JAC, Egger M, Sutton AJ, Egger M, Davey-Smith G, Altman DG (2001). Meta-analysis software. Systematic reviews in health care: meta-analysis in context.

[21] Gerend MA, Shepherd JE (2007). Using message framing to promote acceptance of the human papillomavirus vaccine. Health Psychol.

[22] Hwang Y, Cho H, Sands L, Jeong S (2012). Effects of gain- and loss-framed messages on the sun safety behavior of adolescents: the moderating role of risk perceptions. J Health Psychol.

[23] Consedine NS, Horton D, Magai C, Kukafka R (2007). Breast screening in response to gain, loss, and empowerment framed messages among diverse, low-income women. J Health Care Poor Underserved.

[24] Gallagher KM, Updegraff JA, Rothman AJ, Sims L (2011). Perceived susceptibility to breast cancer moderates the effect of gain- and loss-framed messages on use of screening mammography. Health Psychol.

[25] Jasper JD, Woolf J, Christman SD (2014). Responding to framed health messages: different strokes for different (handedness) folks. Psychol Health.

[26] Keller Pa, Lipkus Im, Rimer Bk (2018). Affect, Framing, and Persuasion. Journal of Marketing Research.

[27] Lee MJ, Kang H (2018). Designing Skin Cancer Prevention Messages: Should We Emphasize Gains or Losses? Message Framing, Risk Type, and Prior Experience. Am J Health Promot.

[28] Lucas T, Hayman LW, Blessman JE, Asabigi K, Novak JM (2016). Gain versus loss-framed messaging and colorectal cancer screening among African Americans: A preliminary examination of perceived racism and culturally targeted dual messaging. Br J Health Psychol.

[29] Mays D, Niaura RS, Evans WD, Hammond D, Luta G, Tercyak KP (2015). Cigarette packaging and health warnings: the impact of plain packaging and message framing on young smokers. Tob Control.

[30] Schneider TR, Salovey P, Apanovitch AM, Pizarro J, McCarthy D, Zullo J, Rothman AJ (2001). The effects of message framing and ethnic targeting on mammography use among low-income women. Health Psychol.

[31] Shen L, Mercer Kollar Lm (2013). Testing Moderators of Message Framing Effect. Communication Research.

[32] Umphrey Lr (2003). The effects of message framing and message processing on testicular self‐examination attitudes and perceived susceptibility. Communication Research Reports.

[33] Voss RP, Corser R, McCormick M, Jasper JD (2018). Influencing health decision-making: A study of colour and message framing. Psychol Health.

[34] Rivers Susan E, Salovey Peter, Pizarro David A, Pizarro Judith, Schneider Tamera R (2005). Message framing and pap test utilization among women attending a community health clinic. J Health Psychol.

[35] Huf S, Kerrison RS, King D, Chadborn T, Richmond A, Cunningham D, Friedman E, Shukla H, Tseng F, Judah G, Darzi A, Vlaev I (2020). Behavioral economics informed message content in text message reminders to improve cervical screening participation: Two pragmatic randomized controlled trials. Prev Med.

[36] Thomas K, Hevey D, Pertl M, Ní CS, Craig A, Maher L (2011). Appearance matters: the frame and focus of health messages influences beliefs about skin cancer. Br J Health Psychol.

[37] Han K, Jo S (2012). Does culture matter?: a cross-national investigation of women's responses to cancer prevention campaigns. Health Care Women Int.

[38] Lin C, Yeh W (2017). How Does Health-Related Advertising with a Regulatory Focus and Goal Framing Affect Attitudes toward Ads and Healthy Behavior Intentions?. Int J Environ Res Public Health.

[39] Tu Y, Lin Y, Fan L, Tsai T, Wang H (2019). Effects of Multimedia Framed Messages on Human Papillomavirus Prevention Among Adolescents. West J Nurs Res.

[40] Shao W (2012). Framing and Efficacy: The Effect of Regulatory Fit on Skin Cancer Prevention and Detection. Journal of Nonprofit and Public Sector Marketing.

[41] Hevey D, Dolan M (2014). Approach/avoidance motivation, message framing and skin cancer prevention: a test of the congruency hypothesis. J Health Psychol.

[42] Cherubini P, Rumiati R, Rossi D, Nigro F, Calabrò A (2006). Improving Attitudes Toward Prostate Examinations by Loss‐Framed Appeals. Journal of Applied Social Psychology.

[43] Kim HJ (2014). The impacts of vicarious illness experience on response to gain- versus loss-framed breast cancer screening (BCS) messages. Health Commun.

[44] Adonis L, Paramanund J, Basu D, Luiz J (2017). Framing preventive care messaging and cervical cancer screening in a health-insured population in South Africa: Implications for population-based communication?. J Health Psychol.

[45] Hoffner C, Ye J (2009). Young adults' responses to news about sunscreen and skin cancer: the role of framing and social comparison. Health Commun.

[46] O'Keefe D, Jensen J (2009). The Relative Persuasiveness of Gain-framed and Loss-framed Messages for Encouraging Disease Detection Behaviors: A Meta analytic Review. Journal of Communication (2).

[47] Webb TL, Sheeran P (2006). Does changing behavioral intentions engender behavior change? A meta-analysis of the experimental evidence. Psychol Bull.

[48] Baumeister RF, Vohs KD, Funder DC (2007). Psychology as the Science of Self-Reports and Finger Movements: Whatever Happened to Actual Behavior?. Perspect Psychol Sci.

