# Supplementary material for: Effects of Message Framing on Cancer Prevention and Detection Behaviors, Intentions, and Attitudes: Systematic Review and Meta-analysis
Source: J Med Internet Res. 2021 Sep 16;23(9):e27634. doi: 10.2196/27634 (PMC8485193; doi:10.2196/27634)
Supplement: Multimedia Appendix 1 [file jmir_v23i9e27634_app1.docx]

**Multimedia Appendix 1. Detailed records of eligible studies (k= 24).**

| First author(year) & country | Funding | Cancer topic | Health behavior:  Prevention OR Detection | Outcomes: Attitude OR Intention OR Behavior | Population: ethnic / Age / Female % | Theory OR Model | Research setting | Delivery channel | Follow-up time | Follow-up channel |
| --- | --- | --- | --- | --- | --- | --- | --- | --- | --- | --- |
| Adonis (2016) South Africa | No | Cervical cancer | Detection | Behavior | South African / 21～65 /100% | Prospect Theory | Online | Email (text only) | 6 months | Health insurance database |
| Cherubini (2005) Italy | No | Prostatic cancer | Detection | Attitude & intention | Northern Italian / 40～65 / 0% | Prospect Theory; Health belief model | Offline | Printed materials (text only) | Real-time | By printed questionnaire |
| Consedine (2007) USA | National Institute of General Medical Science (2SO6 GM54650); National Cancer Institute (1P20 CA 91372 and 1 U54 CA 101388) | Breast cancer | Detection | Behavior | 71% Black and 29% White / 50～70 /100% | No | Online | Telephone | 6 months & 12months | Telephone |
| Gallagher (2011) USA | National Cancer Institute (R03-CA128468); Kent-SUMMA Center | Breast cancer | Detection | Behavior | 48.9% African American and 48.3%Caucasian / 45~85 / 100% | Prospect theory | Offline | Video | 3 months | By telephone or postal mail |
| Gerend (2007) USA | No | Cervical cancer | Prevention | Intention | 85%White; 10% Black or African American; 1% American Indian or Alaska Native; 4% mixed or other / 18~25 /100% | Prospect theory | Offline | Printed materials (text only) | Real-time | By printed questionnaire |
| Han (2012) South Korea | SRC Research Center for Women’s Diseases of Sookmyung Women’s University | Breast cancer | Detection | Attitude & intention | 332 U.S. women, 321 Korean women, and 302 Japanese women / 20~49 /100% | Prospect theory | Online | Web page (text only) | Real-time | By online questionnaire |
| Hevey (2014) Ireland | No | Skin cancer | Prevention | Intention | Irish /16~26 / 59% | Prospect Theory | Offline | Printed materials (text only) | Real-time | By printed questionnaire |
| Huf (study2, 2020) UK | Imperial Health Charity [Ref: 7006/P26U]; Public Health England as a Behavioral Insights Project | Cervical cancer | Detection | Behavior | British / 30~64 / 100% | No | Online | Text message | 12 months | Health and Social Care Information Centre (HSCIC; now ‘NHS Digital’ |
| Hwang (2012) South Korea | National Institutes of Health (CA128438) | Skin cancer | Prevention | Intention | 94.5% white / 12~18 / 45% | Prospect Theory | Offline | Printed materials (text only) | Real-time | By printed questionnaire |
| Jasper (2014) USA | National Science Foundation (SES #0318239 & SES #0620094). | Skin cancer | Prevention | Intention | American / 17~40 / 65% | No | Offline | Printed materials (text only) | Real-time | By printed questionnaire |
| Keller (Exp1,2003) USA | National Cancer Institute (5PO1CA72O99-O3). | Breast cancer | Detection | Intention | 92% white, 3.5% African American, and 2.5% Hispanic / 40~70 / 100% | Hedonic contingency theory | Offline | Printed materials (graphic and textual) | Real-time | By printed questionnaire |
| Keller (Exp2,2003) USA | National Cancer Institute (5PO1CA72O99-O3). | Breast cancer | Detection | Intention | 73% white and 27% African American / 40~70 / 100% | Hedonic contingency theory | Offline | Printed materials (graphic and textual) | Real-time | By printed questionnaire |
| Kim (2014) Singapore | No | Breast cancer | Detection | Attitude & intention | African Americans / 30~71 / 46% | Prospect Theory | Online | Email (video) | Real-time | By online questionnaire |
| Lee (2018) USA | No | Skin cancer | Prevention | Intention | American / 18~32 / 73% | Prospect Theory | Online | Email (text only) | Real-time | By online questionnaire |
| Lin (2017) China | Ministry of Science and Technology, Taiwan (NSC 102-2410-H-024-003) | Colorectal cancer | Prevention | Attitude & intention | Not given / 16~56 / 58.2% | Regulatory focus theory, Theory of planned behavior, Theory of reasoned action | Offline | Printed materials (graphic and textual) | Real-time | By printed questionnaire |
| Lucas (2016) USA | Wayne State University Research Enhancement Program Grant | Colorectal cancer | Detection | Attitude & intention | 132 African Americans and 50 White Americans / 49~65 / 73% | Prospect Theory; Theory of planned behavior | Online | Email (text only) | Real-time | By online questionnaire |
| Mays (2015) USA | American Cancer Society Institutional Research Grant to Georgetown Lombardi Comprehensive Cancer Center (Grant # IRG-97-152-17); the Biostatistics and Bioinformatics Shared Resource of Georgetown Lombardi Comprehensive Cancer Center through Comprehensive Cancer Center Support Grant # P30CA051008 | Lung cancer | Prevention | Intention | 74.9%White, 10.7%black, 14.4% other minority / 18~30 / 44.6% | Prospect Theory | Online | Email (text and image) | Real-time | By online questionnaire |
| Rivers (2005) | National Cancer Institute (R01-CA68427); National Institutes of Mental Health (P01-MH/DA56826); National Institute on Drug Abuse (P50-DA13334) ; Ethel Donaghue Women’s Health Investigator Program at Yale University | Cervical cancer | Detection | Behavior | 59% African American, 27% Hispanic, 11% White, 3% Other / 18~64 / 100% | Prospect Theory | Offline | Video | 6 months | Telephone |
| Schneider (2001) USA | American Cancer Society Grant RPG-93-028-05-PBP; National Cancer Institute Grant R01-CA68427; National Institute of Mental Health Grant P01-MH/DA56826; Ethel Donaghue Women's Health Investigator Program at Yale University | Breast cancer | Detection | Behavior | 43% African American, 27% Anglo, 25% Latina, 2% American Indian, 1% Asian, and 3% from other ethnic groups / 40~91 / 100% | Prospect Theory; Health belief model | Offline | Video | 6 months & 12months | By telephone or by a stamped, preaddressed postcard |
| Shao (2012) Australia | No | Skin cancer | Prevention & detection | Attitude & intention | Not given | Protection-motivation theory | Online | Email (text and image) | Real-time | By online questionnaire |
| Shen (2015) USA | No | Skin cancer | Prevention & detection | Attitude | 81.4% White/Caucasian, 8.8% Asian descent, 2.2% Hispanic descent, 6.4%African descent and 1.1% as other / 18~73 / 69% | Prospect Theory; The theory of reasoned action | Online | Webpage | Real-time | By online questionnaire |
| Thomas (2011) UK | No | Skin cancer | Prevention | Intention | Not given / 16~26 / 58.5% | Prospect Theory; Self-Affirmation Theory | Offline | Printed materials (text only) | Real-time | By printed questionnaire |
| Tu (2018) China | No | Cervical  cancer | Prevention | Attitude & intention | Taiwanese / not given / 100% | The theory of planned behavior | Offline | Video | Real-time | By printed questionnaire |
| Umphrey (2003) USA | No | Testicular cancer | Detection | Attitude | 77% Caucasian, 12.9% Hispanic, 4.3% Asian/Pacific Islander, 1.4% American Indian, 1.4% African American and 2.9%other ethnic / 19~49 / 0 | Prospect Theory | Offline | Printed materials (text only) | Real-time | By printed questionnaire |
| Voss (2018) USA | No | Skin  cancer | Prevention | Intention | American / not given / 66.8% | The Unification Theory (UT); Inclusion/exclusion model (IEM) | Offline | Printed materials (text only) | Real-time | By printed questionnaire |
