# Supplementary figures and images for: Effects of Message Framing on Cancer Prevention and Detection Behaviors, Intentions, and Attitudes: Systematic Review and Meta-analysis
Source: J Med Internet Res. 2021 Sep 16;23(9):e27634. doi: 10.2196/27634 (PMC8485193; doi:10.2196/27634)

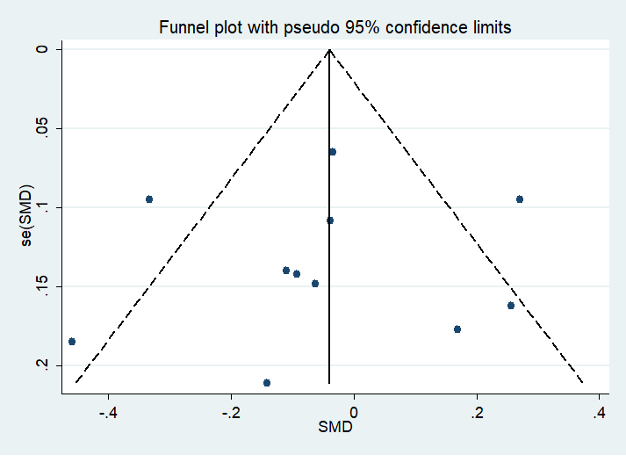

Supplement: Multimedia Appendix 2 [file jmir_v23i9e27634_app2.png]

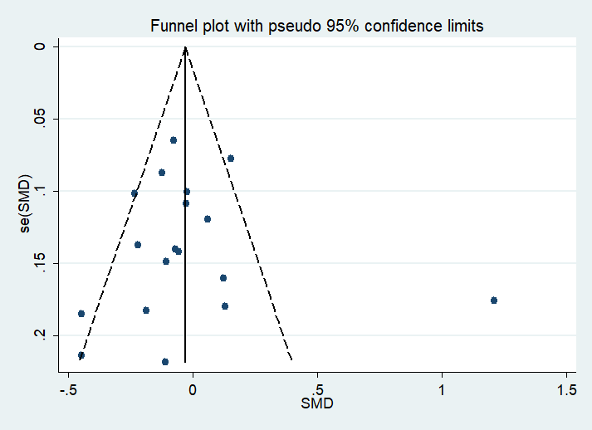

Supplement: Multimedia Appendix 3 [file jmir_v23i9e27634_app3.png]

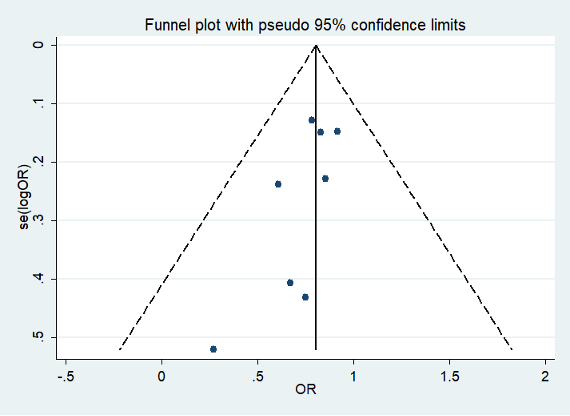

Supplement: Multimedia Appendix 4 [file jmir_v23i9e27634_app4.png]
